# Biotin-responsive basal ganglia disease should be renamed biotin-thiamine-responsive basal ganglia disease: a retrospective review of the clinical, radiological and molecular findings of 18 new cases

**DOI:** 10.1186/1750-1172-8-83

**Published:** 2013-06-06

**Authors:** Majid Alfadhel, Makki Almuntashri, Raafat H Jadah, Fahad A Bashiri, Muhammad Talal Al Rifai, Hisham Al Shalaan, Mohammed Al Balwi, Ahmed Al Rumayan, Wafaa Eyaid, Waleed Al-Twaijri

**Affiliations:** 1 Division of Genetics, Department of Pediatrics, King Abdulaziz Medical City, Riyadh, Saudi Arabia; 2 Department of Radiology, King Abdulaziz Medical City, Riyadh, Saudi Arabia; 3 Division of Neurology, Department of Pediatrics, King Abdulaziz Medical City, Riyadh, Saudi Arabia; 4 Division of Pediatric Neurology, Department of Pediatrics, College of Medicine and King Khalid University Hospital, King Saud University, Riyadh, Saudi Arabia; 5 College of Medicine, King Saud bin Abdulaziz University for Health Sciences, Riyadh, Saudi Arabia; 6 Division of Molecular Pathology and Genetics, Department of Pathology and Laboratory Medicine, King Abdulaziz Medical City, Riyadh, Saudi Arabia; 7 King Abdullah International Medical Research Center, Riyadh, Saudi Arabia

**Keywords:** Biotin-responsive basal ganglia disease, Biotin, Thiamine, SLC19A3, Encephalopathy, Neurometabolic

## Abstract

**Background:**

Biotin-responsive basal ganglia disease (BBGD) is an autosomal recessive neurometabolic disorder. It is characterized by sub acute encephalopathy with confusion, seizure, dysarthria and dystonia following a history of febrile illness. If left untreated with biotin, the disease can progress to severe quadriparesis and even death.

**Method:**

A retrospective chart review of 18 patients with BBGD from two tertiary institutions describing their clinical, magnetic resonance imaging and molecular findings was conducted.

**Result:**

Eighteen children from 13 families seen over a period of nine years (2003–2012) were included. (Age range: 14month to 23 years, M: F: 1:1). The clinical features included sub acute encephalopathy, ataxia (n= 18), seizures (n= 13) dystonia (n=12) ,dysarthria (n= 9), quadriparesis and hyperreflexia (n=9). Magnetic resonance imaging demonstrated abnormal signal intensity with swelling in the basal ganglia during acute crises (n= 13/13) and atrophy of the basal ganglia and necrosis during follow up (n= 13/13). One-third of the present patients showed the recurrence of acute crises while on biotin therapy alone, but after the addition of thiamine, crises did not recur. All of the patients have a homozygous missense mutation in exon 5 of the SLC19A3 gene. The frequency of acute crises, delay in diagnosis and initiation of treatment significantly influenced the outcome. On follow up, four patients died, two had spastic quadriplegia, six had normal outcome and the rest had speech and motor dysfunctions.

**Conclusion:**

Clinicians should suspect BBGD in any child presenting with sub acute encephalopathy, abnormal movement and MRI findings as described above. Both biotin and thiamine are essential for disease management. Since biotin alone could not prevent the recurrence of crises in some patients, a more appropriate term to describe the disease would be biotin-thiamine-responsive basal ganglia disease (BTBGD).

## Background

Biotin-responsive basal ganglia disease (BBGD), also known as thiamine metabolism dysfunction syndrome-2 (THMD2) (MIM: 607483), is an autosomal recessive inherited neurometabolic disorder. BBGD was first described by Ozand et al. in 10 patients, of whom 8 were Saudi, 1 was Syrian and 1 was Yemeni. The disease is characterized by sub acute encephalopathy with confusion, dysarthria and dysphagia with occasional supranuclear facial nerve palsy or external ophthalmoplegia that progresses to severe cogwheel rigidity, dystonia and quadriparesis with a vague history of febrile illness [1]. In 2005, the disease was mapped to chromosome 2q36.3 and was found to result from a mutation in the *SLC19A3* gene that encodes hTHTR2, a second thiamine-transporter [2]. Subsequently, cases have been reported in patients of different ethnicities including those of Lebanese, Portuguese, Indian and Japanese origins [3-6]. Neuroradiological findings include bilateral abnormal signal intensity in the basal ganglia with swelling during acute crises [1,3-6], specifically in the central part of the caudate heads and in part or all of the putamen [1]. Additionally, abnormal signal intensity in the thalami [6], globi pallidi [3], white [1], and brain and cerebellar atrophy [6] are also observed.

BBGD responds to the administration of high doses of biotin (5–10 mg/kg/day) [1,3-6], and the symptoms reappear within 1 month if biotin is discontinued [1]. Although the probands did not respond to thiamine, carnitine, phenobarbital or diazepam treatment in the original report [1], the patients showed improvement with the addition of thiamine to the biotin therapy in another reports [5,6].

Thus far, only 20 cases of BBGD have been described worldwide, and there are anecdotal reports in the literature regarding the natural history of the disease, its neurological outcomes and the genotype-phenotype correlation [1,3-6].

In this study, we report the natural history, genotype-phenotype correlation, biochemical and molecular findings, features on Magnetic Resonance Imaging (MRI) and the importance of thiamine in the treatment regimen of this disease. This large cohort of patients affected with this disease and their results should contribute to the understanding of the natural history and treatment of this devastating neurometabolic disease.

## Method

A retrospective chart review of BBGD patients attending the Biochemical Genetics (Metabolic) and Neurology clinics between 2003 and 2012 was conducted. All BBGD patients from King Abdulaziz Medical City (KAMC) and King Khalid University Hospital (KKUH), Riyadh, Saudi Arabia, were included in the study. The diagnosis of BBGD was confirmed in all living participants and in one deceased individual based on DNA molecular testing. This study was approved by the ethics review boards of all participating institutions.

## Result

In total, 18 patients were included (15 from KAMC and 3 from KKUH). Table 1, Table 2 and Table 3 summarize the demographic information and the results of clinical, molecular and treatment regimens for all patients. All patients were Saudi and the male to female ratio was 1:1. The oldest patient was 23 years old with moderate motor and speech deficits, while the youngest was 15 months old with normal development.

**Table 1:** Demographics, clinical features, long term outcome of the cohort

**#**	**Sex; relative**	**Current age**	**Age of onset**	**Consanguinity**	**Family history**	**C/F at presentatioon**	**Seizure**	**Preceded by history of flu like illness**	**Neurological outcome**	**# of attacks**	**Medications**	**Delayed diagnosis**	**Comments**
1	M	5 yrs	3 and 1/2 yrs	No	No	SAE, ataxia, dysarthria, dystonia	Yes	Yes	Normal	1	Biotin 7 mg/kg/day	No delay	
											Thiamine 200 mg BID		
2	F	15 months	4/12	Yes	No	SAE, Loose mile stones,	No	Yes	Normal	1	Biotin 10 mg/kg/day	No delay	
											Thiamine 200 mg BID		
3	M	4 yrs	1 yr	Yes	No	SAE, loose milestones, dystonia	No	Yes	Normal	1	Biotin 10 mg kg/day Thiamine 200 mg BID	No delay	
4	M ; Brother of patients #5 and 6	10 yrs	7 yrs	Yes	Yes	SAE, ataxia, dystonia, dysarthria	Yes	Yes	Moderate motor and speech deficit	3	Biotin 7 mg/kg/day	No delay	Had recurrence of crises on low dose biotin 2 mg/kg/day. and no recurrence after addition of thiamine and increase the dose of biotin
											Thiamine 100 mg TID		
5	M	23 yrs	2 yrs	Yes	Yes	SAE, ataxia, dysarthria , dystonia	Yes	Yes	Mild motor and speech deficit , drive the car, finish technology college	3	Biotin 5 mg/kg/day	5 years	Had recurrence of crises on Biotin alone and no recurrence after addition of thiamine
											Thiamine 100 mg BID		
6	M	Died at 26 yrs and spastic quadriplegic	4 yrs	Yes	Yes	SAE, ataxia, dysarthria, dystonia	Yes	Yes	Died	6	Biotin 5 mg/kg/day	8 years	With age frequent seizure, had recurrence of crises on biotin alone and no thiamin was added to treatment
7	F	11 yrs	4 yrs	Yes	No	SAE, ataxia, dysarthria, dystonia	Yes	Yes	Moderate motor and speech deficit	2	Biotin 5 mg/kg/day	3/12	Had recurrence of crises on Biotin alone and no recurrence after addition of thiamine
											Thiamine 100 mg BID		
8	F	9 yrs	2 yrs	Yes	Yes	GDD since birth then SAE, ataxia, dysarthria, dystonia	Yes	Yes	Spastic quadriplegic	2	Biotin 5 mg/kg/day	3 years	
											Thiamine 100 mg Tid		
9	M; brother of patients #10 and 11	14 yrs	14 months	Yes	Yes	SAE, Loose mile stones,	Yes	Yes	Mild motor and speech deficit ; 5th grade primary school	3	Biotin 10 mg/kg/day	3/12	Had recurrence of crises on Biotin alone and no recurrence after addition of thiamine
											Thiamine 100 mg TID		
10	F	11 yrs	5 months	Yes	No	SAE, loose milestones, dystonia	No	Yes	Spastic quadriplegic	5	Biotin 10 mg/kg/day	1 year	Had recurrence of crises on Biotin alone and no recurrence after addition of thiamine
											Thiamine 100 mg TID		
11	M	4 yrs	3 yrs	Yes	Yes	SAE, ataxia, dysarthria, loose milestones	Yes	Yes	Died	>10	No treatment	No diagnosis	Retrospectively diagnosed based on diagnosis of his younger sibling.
12	F	4 yrs	3 yrs	Yes	No	SAE, ataxia , loose mile stones	No	Yes	Normal	1	Biotin 10 mg/kg/day	No delay	
											Thiamine 100 mg TID		
13	F	4 yrs	3 yrs	Yes	No	SAE, ataxia, dysarthria, loose mile stones	No	Yes	Normal	1	Biotin 10 mg/kg/day	No delay	
											Thiamine 100 mg TID		
14	M	6 yrs	4 yrs	Yes	No	SAE, ataxia, dysarthria, dystonia	Yes	Yes	Mild motor and speech deficit	3	Biotin 10 mg/kg/day	3/12	Started on this regimen once diagnosed
											Thiamine 100 mg TID		
15	M	Died at 3.5 yrs	3 yrs	Yes	Yes	SAE, ataxia, dysarthria, dystonia	Yes	Yes	Died	1	No treatment	No diagnosis	Retrospectively diagnosed based on diagnosis of his younger sibling.
16	F	13 yrs	11 yrs	Yes	No	SAE, ataxia dysarthria, dystonia	Yes	Yes	Mild speech deficit	3	Biotin 8-10 mg/kg/day	1 and half year	Started on this regimen once diagnosed
											Thiamine 100 mg TID		
17	F; sister of patient #18	2 yrs	2 yrs	Yes	Yes	SAE, ataxia	Yes	No	Normal	1	Biotin 8-10 mg/kg/day	No delay	
											Thiamine 100 mg TID		
18	F	2 yrs and 9 months	2 yrs	Yes	Yes	SAE, ataxia	Yes	Yes	Died	1	No treatment	No diagnosis	Retrospectively diagnosed based on diagnosis of her younger sibling.

*C/F* clinical features, *GDD* global developmental delay, *M* Male, *F* female, *SAE* sub acute encephalopathy.

**Table 2:** Summary of the results

**Result**	**# (%)**
Age of onset 3-4 years	(8/18; 45%)
Consanguinity	17/18 (95%)
One tribe	13/18 (72%)
Saudi	18/18 (100%)
c.1264 A>G (p.T422A) mutation in exon 5 of *SLC19A3* gene	15/15 (100%)
SAE, ataxia triggered by febrile illness	18/18 (100%)
Seizure	13/18 (72%)
Mild-moderate neurological deficit mainly speech and motor	6/18 (33%)
Normal	6/18 (33%)
Spastic quadriplegic	2/18 (11%)
Death	4/18 (22%)

**Table 3:** MRI findings

**Results**	**# (%)**
In acute crises: high signal T_2_ with swelling in basal ganglia (caudate and pautamen) diffuse cortical, subcortical white matters and infratentorial brain.	13/13 (100%)
In chronic follow up: high signal T_2_ in basal ganglia (caudate and pautamen) with atrophy and necrosis	13/13 (100%)
Thalami	4/13 (31%)
Brain stem – all 4 patients had midbrain involvement and one had medulla oblongata as well.	4/13 (31%)
Cerebellum – all 4 patients had cortical involvement, and it was more diffuse in one patient.	4/13 (31%)
Spine – cervical spinal cord	1/13 (7.6%)

### Clinical history and examination

Consanguinity was confirmed in 17 of the 18 (95%) patients. Eight patients were from three families (see Table 1), and 9 of the 18 (50%) patients had a positive family history of a similarly affected sibling. Thirteen of the 18 (72%) patients came from the same tribe.

The age of onset is variable, but onset commonly occurs between 3 and 4 years (45%). All patients presented with the classical clinical features of subacute encephalopathy (confusion, drowsy, altered level of consciousness) and ataxia triggered by febrile illness. Seizures occurred in 13 of the 18 (72%) patients, and the seizures were mainly partial or generalized tonic-clonic seizures. Neurological examination showed: extrapyramidal sign dystonia and/or dysarthria were found in 12 of the 18 (67%) patients. quadriparesis and hyperreflexia in 9 of 18 (50%). Fundus examination were normal in all patients.

### Investigations

**Figure 1:**
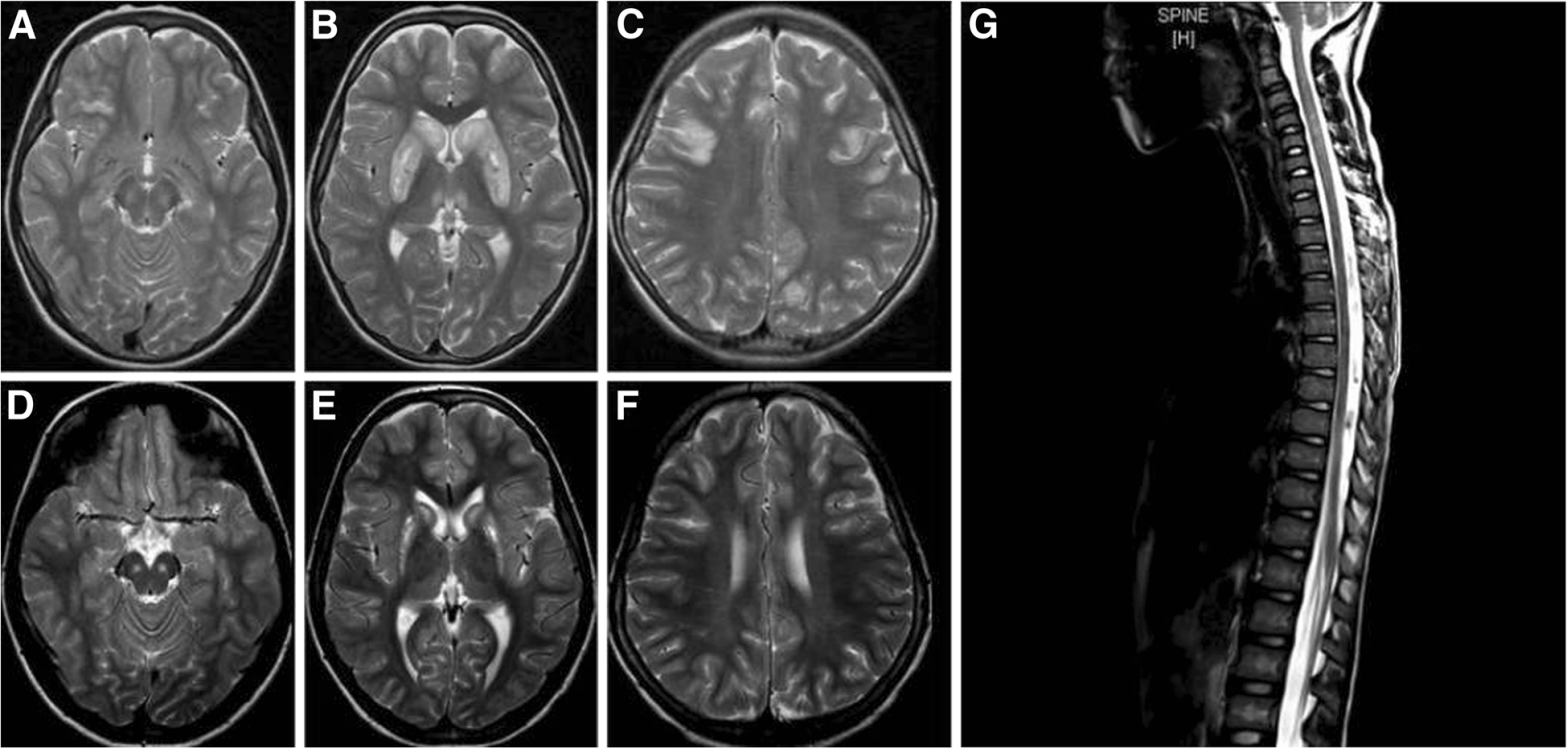
**MRI brain axial T2 weighted spin echo images – (images A to F) and MRI spine sagittal T2 weighted image – (image G).** Upper column: (**A**, **B**, **C**) was done for the patient during the acute crisis. **A**: This image shows high T2 signal and swelling of substantia nigra (at midbrain), and signal abnormality with swelling of right inferior frontal gyrus in the cortical and subcortical region. **B**: It shows high T2 signal and swelling of lentiform, caudate head with small necrotic changes and involvement of medial thalami. **C**: It shows focal high T2 cortical signal and subcortical white matter signal at both frontal and parietal lobes. Lower column: (**D**, **E**, **F**) was done as a follow up exam for the same patient during chronic stage of the disease. **D**: This image was done at the level of the midbrain and shows focal high T2 fluid signal in the substantia nigra which evolved from swelling seen during the acute crisis (image A). E: It shows high T2 (fluid signal) and atrophy in the basal ganglia (lentiform and caudate nuclei) indicates evolved swelling into necrosis. **F**: It shows interval resolution of the cortical and subcortical signal seen during the acute crisis at the frontal lobes. MRI Image of the spine (**G**) shows patchy high T2 intramedullary signal most conspicuous in the cervical spinal cord with swelling extends from C3 to C7 vertebral level.

All of the following were unremarkable which includes: lactate level in blood and CSF, ammonia, biotinidase, creatine phosphokinase, acylcarnitine profile, copper, ceruloplasmin, blood for biotin and thiamine level, plasma aminoa acids, urine for amino acids, urine for organic acids, liver enzymes, coagulation profile, lipid profile and cerebrospinal fluid for cell counts, protein, glucose and culture. Magnetic resonance imaging findings (see Figure 1) consisted of abnormal signal intensity at the caudate and putamen with diffuse involvement of the brain including the cortical and subcortical white matter and the infratentorial region, while atrophy and necrosis of the basal ganglia was demonstrated in all patients who had MRIs during chronic follow-up. Cerebellar, thalami and brain stem involvement were confirmed in 4 of the 13 (31%) patients, while one patient had a spinal lesion with increased T2 signal intensity, particularly in the cervical region. Fifteen of the 18 (83%) patients were diagnosed based on DNA molecular testing, and all were found to have the same homozygous missense mutation in exon 5 of the *SLC19A3* gene (Table 2); their parents were heterozygous for this mutation. The remaining three patients were diagnosed retrospectively after the diagnosis of their younger siblings.

### Follow up and outcome

In 8 of the 18 (45%) patients, the diagnosis was delayed, while immediate diagnosis was achieved in 7 individuals.

Four of the 18 (22%) patients died. Three patients died because treatment was not initiated due to lack of diagnosis. One patient who was diagnosed died at 26 years of age following an acute crisis; he had been receiving biotin therapy for 18 years. One-third of the patients showed mild to moderate neurological deficit, mainly in speech and motor function; one-third of the patients were normal; and two patients had severe global neurological deficits and spastic quadriplegia. In general, the cognitive outcome is better than speech and motor outcomes. All diagnosed patients improved with biotin (5–10 mg/kg/day) therapy while alive. The maximum dose of biotin was used 12 mg/kg/day, the dose schedule range from 1–3 times daily with no consistent regimen and the tablet strength was 10mg tablet. Six of the 18 (33%) patients had a recurrence of acute crises while on biotin alone, but after the addition of thiamine, there were no recurrences of those crises.

## Discussion

In this study, we report the largest cohort to date of patients with BBGD. Our results support the findings of previous reports regarding the classical clinical presentation, the high prevalence of this disease in Saudi Arabia compared to other countries, the deterioration and death of patients who do not receive biotin treatment, autosomal recessive inheritance, the importance of biotin in treatment regimens and the cause of the disease in a mutation of the *SLC19A3* gene, which was found in all patients [1,3-6]. Our study demonstrated the following important points:

### The natural history and the dosage regimens for biotin and/or thiamine in BBGD patients are incompletely understood

Previous reports showed highly variable neurological outcomes even among siblings from the same family [1,3-6]. Ozand et al. reported that 5 of 10 (50%) patients with BBGD were normal with 5–10 mg/kg/day of biotin over a variable period of follow-up ranging from 5 to 10 years [1], while 5 mg/kg/day of biotin was ineffective in another report, and the patient was bedridden [6]. Interestingly, one BBGD child responded well to lower dosages of biotin (5–30 mg/day) [3]. Furthermore, Debs et al. reported on two siblings with BBGD, a 33 year old and a 29 year old, both had the same mutation. The man responded well to biotin alone, while the woman did not improve until thiamine was added to the treatment regimen. This significant heterogeneity demonstrates the complexity and ambiguity of the natural history and treatment of BBGD. Furthermore, this cohort demonstrated intrafamilial heterogeneity as we observe different outcomes within siblings of the same family, same environments and same molecular findings (see Table 1). However, the favorable outcome could be related to early initiation of treatment in several of them.

### Thiamine and biotin are essential in the treatment regimens of BBGD

In original reports, thiamine was not effective [1]. However, one-third of the patients reported in this cohort showed recurrence of acute crises while on biotin alone, but with the addition of thiamine, the recurrence of these crises was prevented. This result supports the hypothesis that the impairment of thiamine transport in the brain has a critical role in this disorder. Furthermore, the *SLC19A3* gene encodes hTHTR2, a second thiamine-transporter and not a biotin transporter [2]. In fact, hTHTR2 demonstrates 48% structural identity to hTHTR1, which is also a thiamine transporter, while demonstrating only 17% identity with hSMVT, which is a known biotin transporter [7]. Additionally, Subramanian et al. proved that biotin is not a substrate for hTHTR2 [7]. This evidence supports the importance of thiamine in the treatment of this disease but leaves the mechanism by which BBGD patients improved dramatically on biotin, as we observed in 100% of our cohort, unclear. However, it could be hypothesized that the biotin and thiamine transporters in the basal ganglia are closely associated and thus act synergistically [7]. Therefore, we recommend that the treatment regimen for patients with BBGD contain both thiamine and biotin.

### Factors contributing to clinical outcome

The gap between the age of disease onset and the date of diagnosis correlates directly with the neurological outcome. Eight of the 18 patients who had a delayed diagnosis displayed mild to moderate neurological deficits, while seven patients who were diagnosed immediately achieved normal development. Another important factor is the number of attacks of acute crises, as a poor outcome is directly associated with more acute crises in the present patients. All those children with normal outcome had only one event. Additionally, the immediate treatment with biotin and thiamine is an important aspect that contributes to normal outcomes, as reported cohort showed no recurrence of crises on this regimen for a period of up to 5 years of follow-up. However, it is very difficult to extrapolate firm conclusion with regards to aforementioned factors as the median follow up of normal children were 16 months (1 month-3 year) which is too short compare to poorer outcome individuals.

### Magnetic resonance imaging commonly shows an effect in the basal ganglia and could involve other parts of the brain and spinal cord

In the original report by Ozand et al., the MRI findings consist of bilateral necrosis in the basal ganglia, particularly at the central part of the caudate heads and part or all of the putamen with severe edema during the acute crisis in addition to white matter involvement at the grey–white matter junction [1]. Subsequent reports confirmed these findings and added the involvement of the globi pallidi, thalami, cerebellum and brain stem [3,6]. Our largest cohort demonstrated these findings and showed abnormal signal intensity and swelling at the caudate and pautamen with diffuse involvement of the brain including the cortical, subcortical white matter and infratentorial region, while atrophy and necrosis of the basal ganglia appeared during chronic follow-up. Cerebellar, thalami and brain stem involvement are found in one-third of patients (see Figure 1). Interestingly, one patient demonstrated spinal cord involvement with increased T_2_ signal intensity particularly at the cervical region, which was not reported previously. This could be easily explained by the fact that previous patients did not undergo MRI of the spine, and neither did the patients in this report. It would be valuable to perform spinal MRI for all patients affected with this disease to see the extent of this finding.

### The genotype-phenotype correlation is poor

All current patients have a homozygous missense mutation in exon 5 of the SLC19A3 gene [c.1264 A>G (p.T422A)]. This mutation is a founder mutation in Saudi families, because all of the patients of Saudi origin reported as having BBGD also carry this mutation. Other reported mutations in the SLC19A3 gene include a homozygous missense mutation in exon 5 of the gene [c.68 G>T (p.G23 V)] in a Yemeni family; a homozygous missense mutation in exon 3 of the gene [c.958 G>C (p.E320Q0] reported in four Japanese patients who are biotin unresponsive; a frame shift mutation in exon 2 [c.74dupT(p.Ser26 LeufsX19)]; and an intron mutation (c.980-14 A>G), which was discovered in 2 siblings in a heterozygous state of European ancestry. Despite having the same mutation, children from the same family have extremely variable outcomes, ranging from normal to severely handicapped [1-3,5,6].

### Future directions

Despite its discovery 14 years ago, several questions regarding BBGD remain unanswered. Animal models are vital to understanding the pathophysiology of thiamine and biotin transport in the brain, particularly the levels of biotin and thiamine in the brain at the basal ganglia. This information is critical to understanding the clinical picture, complications and management of this disease. Moreover, investigators should make an effort to find a biomarker for this disease, which will help to accurately determine the appropriate dosages of biotin and thiamine to consequently improve the outcome of the disease. Finally, diagnosing more asymptomatic cases through the screening of high risk tribes and international registries to accommodate more cases of the *SLC19A3* gene defect will lead to a better understanding of the natural history of the disease and will facilitate well designed treatment and outcome studies.

## Conclusion

Based on the evidence obtained from our cohort, we recommend changing the name BBGD to biotin-thiamine-responsive basal ganglia disease (BTBGD). Clinicians should consider this disorder in any patients with subacute/acute encephalopathy, ataxia triggered by febrile illness and basal ganglia involvement.

## Competing interests

The authors declare that they have no competing interests.

## Authors’ contributions

MAF performed the majority of work associated with preparing, writing and submitting the manuscript and contributed to the clinical diagnosis and management of the patients from King Abdulaziz Medical City. RJ summarized the clinical data and contributed to the management of the patients from King Abdulaziz Medical City. FAB summarized the clinical data and contributed to the diagnosis and management of the patients from King Khalid University Hospital. TAR edited the manuscript and contributed to the clinical diagnosis and management of the patients from King Abdulaziz Medical City. MAM and HAS assessed and described the radiological findings obtained from the patients. AAR, WAT and WE edited the manuscript and contributed to the clinical diagnosis and management of the patients from King Abdulaziz Medical City. MAB edited the manuscript and summarized the molecular genetics data for the patients. All authors read and approved the final manuscript.
